# Overcoming Antibiotic Resistance with Novel Paradigms of Antibiotic Selection

**DOI:** 10.3390/microorganisms10122383

**Published:** 2022-11-30

**Authors:** George Tetz, Victor Tetz

**Affiliations:** Human Microbiology Institute, New York, NY 100141, USA

**Keywords:** antibiotic resistance, bacterial pathogens, antimicrobial susceptibility tests, persisters, collective antibiotic resistance, biofilm, TezR

## Abstract

Conventional antimicrobial susceptibility tests, including phenotypic and genotypic methods, are insufficiently accurate and frequently fail to identify effective antibiotics. These methods predominantly select therapies based on the antibiotic response of only the lead bacterial pathogen within pure bacterial culture. However, this neglects the fact that, in the majority of human infections, the lead bacterial pathogens are present as a part of multispecies communities that modulate the response of these lead pathogens to antibiotics and that multiple pathogens can contribute to the infection simultaneously. This discrepancy is a major cause of the failure of antimicrobial susceptibility tests to detect antibiotics that are effective in vivo. This review article provides a comprehensive overview of the factors that are missed by conventional antimicrobial susceptibility tests and it explains how accounting for these methods can aid the development of novel diagnostic approaches.

## 1. Background of the Problem

Despite the availability of numerous antibiotics and many types of antimicrobial susceptibility tests (ASTs), over 5,000,000 people die each year due to bacterial infections [1]. According to the World Health Organization, antimicrobial resistance will become one of the leading causes of death worldwide within the next 30 years [2]. Although the spread of antibiotic resistance plays an important role in these devastating diseases, it is not the primary cause of these deaths. Indeed, cases of pan-resistant bacteria are still rare, and a number of therapeutic options exist for the treatment of multidrug-resistant bacteria [3]. Instead, the main problem is a lack of effective and appropriately designed antimicrobial susceptibility testing methods.

Numerous studies have demonstrated a lack of a relationship between the results of routine ASTs and responses to selected antibiotics, indicating that these ASTs were unable to predict the clinical response to antibiotics [4,5]. These clinical observations have given rise to the “90–60” rule: “susceptible” infections respond well to appropriate therapy in 90% of cases, whereas “resistant” infections respond well to these antibiotics in 60% of cases [6,7,8].

This is also clearly reflected in official statistics that show that around 32,000 people die annually in the US because of antibiotic-resistant bacteria [9]. To put this in perspective, over 50,000 patients with ventilator-associated pneumonia and 270,000 with sepsis die annually in the US, highlighting that antibiotic-resistant bacteria are not the only cause of death when it comes to deadly bacterial infections [10,11,12].

In this review, we analyze the reasons why available ASTs directed to select antibiotics against non-blood-borne infections, such as respiratory infections, skin and soft tissue infections, and urinary tract infections, fail to select effective antibiotics. We also analyze emerging methods of antibiotic selection that can fix the fundamental flaws of routine ASTs.

## 2. Overview of Conventional Antimicrobial Susceptibility Tests

### Culture-Based Antimicrobial Susceptibility Tests

Culture-based ASTs are based on the response of the lead bacterial pathogens from biosamples to antibiotic treatment and are considered a primary diagnostic test for antibiotic selection in clinical settings. In accordance with current recommendations, pure culture isolates should first be obtained and used to test antibiotic efficacy [13]. Biosamples such as sputum, bronchoalveolar lavage, wound swabs, and urine are processed in order to isolate a pure bacterial culture of the lead pathogen. This can be achieved by cultivating in selective or non-selective solid or liquid nutrient media. The isolation of pure bacterial cultures is a time-consuming step that requires 24–72 h depending on the nature of the lead pathogen [14]. Subsequently, the sensitivity of the lead pathogen to different antibiotics is assessed. These methods rely on measuring the inhibition of bacterial growth in the presence of different pre-determined concentrations of antibiotics over time (usually 24–72 h) using a variety of methods, such as the microdilution method, the disk diffusion method, and E-tests [15]. Culture-based ASTs are slow, and it can take up to five days for a doctor to receive a suggestion regarding which antibiotic they should select to treat their patient [16].

Therefore, in order to accelerate the growth-dependent AST step, automated tests have been implemented that require the isolation of a pure bacterial culture, thus replacing the need for a second step of long cultivation of primary pathogens in the presence of different antibiotics [13,17,18,19,20]. Moreover, automated assays can provide more reliable and precise results. Examples of the most widely used automation methods are those that enable the preparation of two-fold dilutions of antibiotics in a liquid growth medium with accelerated and more accurate readout [21]. Alternatively, they can be based on novel methods such as fluorescence-based or MALDI-TOF mass spectrometry technologies [22].

In recent years, some emerging AST techniques have been developed that evaluate very early alterations in different parameters of bacterial cells in the presence of antibiotics. These methods study the morphological changes in individual bacterial cells induced by antibiotics and include the analysis of parameters such as reduction of the early speed growth, alterations of metabolic status, nanomotion detection, identification of resistance-associated macromolecules, detection of growth-related molecules, and antibiotic degradation products, frequently combining optical-based ASTs with some novel approaches, such as micro-channel resonators [20,23,24,25].

Microbiological analysis of data from these growth-based AST includes the evaluation of the minimum inhibitory concentrations (MICs) of tested antimicrobials and the categorization of bacteria as “susceptible” or “resistant” by applying accepted breakpoint values and estimated thresholds of the tested antibiotics to these MICs [26].

However, all of these automated applications still require pure bacterial isolates obtained through routine culture and do not change the phenotypic AST principle. Although these novel methods claim to conduct ASTs sometimes within the hour, such statements do not reflect the need for another 24–72 h cultivation to obtain pure bacterial cultures.

Since patients with serious bacterial infections require the initiation of antibiotic therapy in less than 8 h, these culture-based ASTs cannot replace empirical antibiotic selection that apparently requires diagnostic guidance since it fails to select effective antibiotics in over 50% of cases [13,27]. Even after an hour, patients with severe infections are left without effective antibiotic therapy, which significantly reduces the chance of a positive outcome and increases mortality rates [27,28].

However, the slow turnaround of these methods that take days to get results is not the only contributor to bacterial-infection-related mortality, since even after long cultivation, culture-based ASTs frequently fail to select effective antibiotics [13]. One of the most notable demonstrations of the failure of culture-based ASTs to select effective antibiotic treatments is the persistence of infections and recurrent infections that cannot be eradicated with AST-guided antibiotic therapy [8]. Despite these shortcomings, culture-based ASTs remain clinicians’ primary method for antibiotic selection.

## 3. Overview of Methods That Detect Genes Conferring Antibiotic Resistance

As stated above, it is critical to treat patients with severe bacterial infections with effective antibiotics as soon as possible. This explains the rapid rise in diagnostics based on the identification of genomic signatures that predict antimicrobial resistance [29]. These methods do not always require the isolation of pure bacterial cultures and can directly determine the presence of ARG within biosamples [30].

ARG detection can be performed using different methods such as PCR, sequencing, and transcriptome analysis to identify the presence of genes conferring antimicrobial resistance or certain mutations [31]. For the more recently introduced nucleic acid amplification technology (NAAT), the presence and expression of ARGs were determined after pre-incubation of the bacterial probe with a particular antibiotic [32]. Although molecular methods for ARG detection usually do not require time-consuming steps—such as the isolation of pure cultures—they cannot provide satisfactory evidence of antibiotic resistance and are frequently viewed as surrogates for culture-based ASTs [33].

The primary issue with ARG detection methods is that the presence of resistance genes does not always confer phenotypic resistance [34]. Firstly, resistant genes may not be expressed. Secondly, another issue with ARG detection methods is that the identified genes may not be related to the pathogens that cause the disease, and that the resistance genes may not be functional [35,36]. Moreover, not all ARGs are known, and the functional expression levels of different ARGs vary for different bacteria [35,36,37,38].

The most broadly used PCR-based methods largely fail to account for adaptive antibiotic resistance, such as the work of efflux pumps or porin loss, while requiring trained personnel and expensive equipment [34,37].

As a result, antibiotics selected with molecular ARG detection tests are frequently ineffective and tend to be unnecessarily broad-spectrum, which is contrary to the concept of antimicrobial stewardship, which aims to diminish unnecessary exposure to broad-spectrum antibiotics [38,39].

In summary, both conventional culture-based ASTs and methods based on ARG detection frequently fail to select appropriate antimicrobials, and the antibiotics selected by these methods fail to be effective when used in patients.

## 4. Mechanisms Underlying Failure of Conventional Antimicrobial Susceptibility Tests

Below, we review the fundamental flaws of routine culture-based and genetic ASTs that result in failure to select effective antibiotics.

### 4.1. General Considerations of Minimum Inhibitory Concentrations and Thresholds for Non-Blood Borne Infections

Routine culture-based ASTs categorize pathogens as “susceptible” or “resistant” by comparing minimal inhibitory concentration (MIC) values that should be below thresholds predetermined for each antibiotic [40]. The acceptance of these thresholds is based on a comparison of the MIC and plasma pharmacokinetics of each anti-infective agent [5]. However, these thresholds do not consider the particularities of antibiotic penetration into different organs and tissues [5,7,8].

Moreover, since only some of the unbound antimicrobial agents are capable of providing antibacterial effects, the particularities of binding of antibiotics with proteins and other elements or their penetration into cells in different tissues would differ from those in plasma [41]. For example, the difference between the free concentration of the same antibiotic would differ significantly between the epithelial lining fluid (if the target site is the lung), the urine for a urinary tract infection, soft tissues for skin and soft tissue infections, and the blood [42,43,44,45]. However, conventional ASTs categorize the susceptibility of bacteria isolated from biosamples of any tissue based on thresholds relying on pharmacokinetics and pharmacodynamics (PK/PD) of the tested antibiotics in blood [20,42]. One of the most striking examples is Fosfomycin, whose MIC and zone diameter breakpoint, for a majority of pathogens according to the latest EUCAST and CLSI criteria, are considered below 64 mg/L; however, its peak urine concentration can be over 4000 mg/L and remains at over 100 µg/mL for 48 h after a therapeutic dose [43,44].

Therefore, for non-blood-borne infections, determination of antibiotic efficacy using antimicrobial agents at the free and unbound concentrations that can be achieved at the particular side of infection and not the total plasma concentration seems to be more rational [42].

Another issue is that conventional AST methods study the efficacy of antibiotics based on a fixed concentration that remains stable throughout the cultivation period. However, because different antibiotics possess different mechanisms of action, their efficacy depends on more complicated parameters than just the mean plasma concentration. All antibiotics can be divided into three major groups: time-dependent, concentration-dependent, and dependent on total drug exposure with the area under the curve (AUC) [45,46].

For example, the efficacy of penicillins, cephalosporins, and carbapenems depends on the time during which they contact bacteria with a concentration above the MIC (T > MIC) that is set by the AST in question [47]. However, the efficacy of fluoroquinolones, aminoglycosides, and polymyxins correlates best with their maximum peak concentrations (Cmax/MIC) at the site of infection because they display concentration-dependent effects [48]. Glycoside antibiotics are both concentration- and time-dependent (AUC/MIC) [48,49]. Neither Cmax/MIC nor AUC/MIC is considered by existing ASTs, since they do not account for pharmacokinetic parameters [47]. The lack of consideration of these particularities by ASTs misleads the selection of an appropriate therapy with both over- and underestimation of antibiotic efficacy [50].

Thus, testing antibiotics while considering T > MIC, Cmax/MIC, and AUC/MIC, depending on the class of antibiotics, would provide more accurate results and enable more precise therapy selection.

### 4.2. Biofilms and Bacterial Multicellularity Are Not Considered by Conventional Antimicrobial Susceptibility Tests

Standard ASTs evaluate antibiotic efficacy either in planktonic growing bacteria or in the form of monobacterial lawns, but not in bacterial biofilms [51,52,53]. However, recent studies have shown that bacterial biofilms play an important role in certain respiratory tract, urinary tract, and skin and soft tissue infections, as biofilm formation is one of the most effective survival strategies for bacteria in any ecological niche [54,55,56,57,58,59,60].

Bacterial biofilms are complex bacterial structures in which cells are covered with a surface bilipid membrane-like structure and embedded in an extracellular polymeric matrix composed of bacterial secreted proteins, extracellular nucleic acids, and polysaccharides [61,62,63,64]. Together, biofilm surface membrane-like structures decrease antibiotic penetration and biofilm matrix by binding and inactivating antibiotics, protecting bacteria within biofilms against environmental assaults, including those posed by antimicrobial agents [65]. Thus, bacteria within biofilms are known to be more than up to 1000 times less sensitive to antibiotics when compared to their planktonic counterparts [66].

Another notable element of the higher tolerance of bacteria in biofilms is that microorganisms within these microbial communities share multicellular properties [67,68,69]. 

Bacteria in biofilms are characterized by the formation of subpopulations with diverse metabolic activities [70]. Certain cell subpopulations are characterized by low metabolism, which in turn contributes to antibiotic tolerance owing to the inactivity of antibiotic targets [71]. There are multiple routes by which low metabolism prevents antimicrobial action. For example, low bacterial protein or DNA synthesis reduces the effectiveness of protein synthesis inhibitors and quinolones, respectively [72]. Alternatively, reduced peptidoglycan production bypasses the antibacterial activity of β-lactams [71]. Other types of antibiotic tolerance are realized by the activation of stress responses in biofilms, including those due to nutrient or oxygen limitation [73,74].

Finally, especially significant within biofilms is formation of a subpopulation of dormant persister cells that are particularly tolerant to antibiotics [75]. The fraction of persister cells in biofilms is 1/10,000, and they have some features that are unique to the more broadly found subpopulations of metabolically impaired bacteria [76]. The biggest challenge of persisters cells in antibiotic treatment failure is that once the concentration of antibiotic drops, surviving persisters can reconstitute the biofilm, resulting in a relapsing and recurrent infection [77,78].

Along with the inhibition of antibiotic penetration and inactivation, biofilms contribute to other strategies for bacterial survival in the presence of antibiotics. Biofilm-type growth impairs DNA repair and mismatch repair systems, which in turn triggers the mutS, mutL, uvrD, and oxoG resistance component (MutT, MutY, and MutM) pathways to react to various antibiotics [79,80].

One of the elements of multicellularity is realized through a particular intermicrobial communication strategy involving chemical signaling via quorum sensing (QS) [81]. QS bacteria synthesize, secrete, and detect specific signaling molecules called autoinducers. Both Gram-negative and Gram-positive bacteria use QC for interbacterial communication. However, different types of bacteria use different autoinducers, with Gram-negative bacteria mainly using N-acyl homoserine lactone and Gram-positive bacteria using a particular QS peptide [82].

Originally, QS was believed to only control bacterial density in biofilms and, along with its increase, trigger biofilm dispersal through the inhibition of matrix compound synthesis, increased production of surfactants, or the degradation of extracellular polymeric substances [83]. However, recent studies have shown that QS modulates antibiotic resistance of biofilm-localized bacteria [84]. One of the mechanisms by which this occurs is through the regulation of bacterial efflux pumps when QS signaling upregulates the overexpression of efflux pump genes [85,86,87]. Some studies have shown that autoinducers can result in overproduction of the MexAB-OprM efflux pump, which plays a significant role in multidrug resistance in *P. aeruginosa*, rendering resistance to aztreonam, gentamicin, tetracycline, quinolones, β-lactams, and tobramycin [88,89,90].

Another example of how QS aids bacteria in withstanding antibiotic assault has been shown in *Escherichia coli* [91]. For instance, overexpression of SdiA, a LuxR homologue of QS regulators of *E. coli* density, regulates the expression of the AcrAB protein, a component of the multidrug-resistant antibiotic efflux pump AcrAB-TolC [92,93,94].

In addition to biofilms, microbial communities formed by spore-forming bacteria of polymicrobial communities having “sporobiota” members are also particularly resistant to antimicrobial therapy [95,96]. Spore-forming bacteria are characterized by a unique tolerance to antibiotics because of the presence of endospores that are practically impermeable to antibiotics due to having a highly resistant and impenetrable core, cortex, and coat as well as uniquely-structured membranes [97,98]. In clinical practice, infections associated with spore-forming bacteria are characterized by a high rate of recurrence and relapse after the death of vegetative cells due to antibiotic assault because spores allow for bacterial regrowth [99,100,101]. The formation of spores by spore-forming bacteria varies depending on the biofilm and non-biofilm modes of growth, as well as between mono- and polymicrobial communities. Since neither of these features is considered by routine ASTs, they are unable to accurately account for the particularities of sporulation, resulting in ineffective antibiotic selection.

In summary, the lack of consideration of biofilm growth by conventional ASTs results in numerous pitfalls of antibiotic selection.

### 4.3. Antibiotic Resistance within Multispecies Communities Is Not Considered by Conventional Antimicrobial Susceptibility Tests

Conventional culture-based methods study the antibiotic response of a lead bacterial pathogen in pure bacterial culture, despite the fact that the majority of infections are polymicrobial [102,103,104,105]. The whole concept of the “lead pathogen” reflects an outdated belief that only pathogens having particular virulent factors contribute to disease; however, it neglects modern observations that other bacteria, even those lacking critical virulence factors, also play a crucial role in infection and antibiotic resistance [106,107].

Therefore, routine ASTs fail to consider interspecies interactions that have a profound effect on the efficacy of selected antibiotics [108]. The resistance mechanisms observed within polymicrobial communities are broad, providing resistance to β-lactams, aminoglycosides, chloramphenicol, and other antibiotics.

There are three major ways in which mixed bacterial communities contribute to protection from antibiotic assault, although they are frequently not mutually exclusive and can also be realized through the formation of microbial biofilms and QS [109].

The first is collective antibiotic resistance, when antibiotic resistance factors released by one type of bacteria are shared across different species, protecting and increasing the MIC of the entire community [109,110,111]. There are several mechanisms by which one member of a polymicrobial community exhibiting antibiotic resistance can protect its cohabitants, even those that do not exhibit antibiotic resistance patterns. One of these ways is realized by the secretion of enzymatic factors directly into the outer environment and especially into the biofilm matrix [112]. For example, β-lactamases secreted by β-lactamase-producing *E. coli* trigger extracellular β-lactam degradation, protecting *Salmonella* spp. and enabling them to survive at antibiotic concentrations above the MIC observed in a pure culture of *Salmonella* spp. [112]. Another way is through the release of membrane vesicles (MVs) [113]. MVs are nanostructures that contain nucleic acids and proteins, and which are widely distributed in different microbial communities. For example, MVs secreted by β-lactamase-producing *M. catarrhalis* hydrolyze β-lactam antibiotics and protect cohabitant *Streptococcus* spp. and *Haemophilus influenzae* within mixed biofilms [114,115]. Another notable example occurs during *Stenotrophomonas maltophilia* infection, in which MVs packed with β-lactamases are secreted within a multispecies community, protecting β-lactam-sensitive *Pseudomonas aeruginosa* and *Burkholderia cenocepacia* by dramatically increasing their MICs for imipenem and ticarcillin [116]. Finally, some factors that inhibit antibiotics and protect the entire population are released due to so-called “altruistic death” [117]. This occurs when resistant bacteria protect vulnerable cohabitats with substances released from these resistant bacteria upon their lysis, thus making their cell death beneficial for the whole community [118]. Notably, recent research has shown that bacteria that are present within a polymicrobial community, even at a very small fraction (0.05% of the whole population), can significantly modulate the synthetic activity of other cells in the community, promoting a collective antibiotic response [119].

The second way in which mixed microbial communities protect against assault by antibiotics is by collective tolerance to antibiotics, which occurs when certain bacteria slow down their metabolism, resulting in a slower rate of bacterial death [120]. The third way is antimicrobial exposure protection, where sensitive members within a community are protected by other co-habitants of the multispecies community by reducing the concentration of antibiotic exposure [121].

Moreover, along with the protection of sensitive cohabitants by the inactivation or binding of antibiotics, there is an increasing body of evidence that, within polymicrobial communities, different bacterial species modulate the expression of antibiotic resistance genes [108,122,123,124,125]. Briaud et al. showed that *S. aureus* coexistence with *P. aeruginosa* resulted in the upregulation of several antibiotic pumps belonging to the Nor family, leading to an increase in *S. aureus* resistance to ciprofloxacin and tetracycline [123]. However, some antibacterial effects could be enhanced by co-culture with the same bacterial species. Thus, the antimicrobial activity of norfloxacin against *S. aureus* is increased because exposure to *P. aeruginosa* exoproducts enhances the intracellular accumulation of norfloxacin by *S. aureus* [125].

The third way for the unique profile of antibiotic resistance in microbial communities was shown in a recent study that examined the effects of the species-specific antibiotics colistin and fusidic acid on *P. aeruginosa* and *S. aureus* in a multispecies population [108]. The results showed that the polymicrobial environment involves both non-heritable and heritable mechanisms of antibiotic resistance [108]. Genome sequencing confirmed that the coculture of *S. aureus* and *P. aeruginosa* increased the frequency of colistin-resistant *P. aeruginosa* isolate formation, triggering the formation of single nucleotide polymorphisms in genes involved in lipopolysaccharide or pilin biosynthesis, thereby creating a form of heritable resistance against this antibiotic.

Along with multiple ways of collective protection of different species within polymicrobial communities against antibiotics, Galera-Laporta et al. showed in a recent study that the population response to some antibiotics in multispecies communities can be opposite to that of each bacterium when cultured as a pure bacterial culture [126]. Single-species *E. coli* was sensitive to ampicillin, while single-species *Bacillus subtilis* was tolerant to ampicillin. However, when the two organisms coexisted in coculture, *E. coli* could grow in the presence of the antibiotic, while *B. subtilis* could not.

The lack of recapturing of multispecies communities by conventional ASTs that rely on antibiotic selection in monopathogen settings also results in pitfalls of antibiotic selection due to a lack of consideration of mutualistic interactions. Indeed, within mixed biofilms, different bacteria support each other, minimizing energy loss by sharing different nutritional factors produced by other microorganisms [127]. Therefore, the ability of different bacteria, including lead bacterial pathogens, to survive within multispecies communities also relies on the existence and activity of other “supporting bacteria” or “accessory pathogens” [107]. The killing of these bacteria by antibiotics results in deprivation of the lead bacterial pathogens, making them more sensitive to the negative assaults of the outer environment.

In summary, traditional ASTs are based on unimicrobial bacterial cultures that do not take into account complicated interbacterial interactions arising from polymicrobial growth and the presence of cohabiting species that confer collective protection of the microbial community, resulting in both false-positive and false-negative therapy selection [108].

### 4.4. Bacterial Regulation through Teazeled Receptors Is Not Considered by Conventional Antimicrobial Susceptibility Tests

A novel universal signaling and regulatory receptor system was recently discovered that involves reverse transcriptases, integrases, and previously unknown DNA- and RNA-based receptors located outside the cell membrane called Teazeled receptors (TezRs) [128,129]. This system plays a unique role in sensing, remembering, and regulating cell responses to various chemical, physical, and biological stimuli. The sensory and regulatory functions of TezRs control all major aspects of bacterial behavior, such as cell growth, biofilm formation and dispersal, nutrient utilization, virulence, chemo- and magnetoreception, responses to external factors (e.g., temperature, UV, light and gas content, and chemicals), recombination, and mutations. In addition, they supervise the functions of other bacterial receptor-mediated signaling pathways. Importantly, TezRs are responsible for the formation and maintenance of bacterial cell memory, as well as the ability to “forget” preceding events.

TezRs are classified based on the structural features of their DNA- or RNA-containing domains as well as based on their association with the membrane. Primary TezRs are those that are more tightly bound to the cell membrane, while secondary TezRs can be easily washed out along with the culture medium or matrix. Since both primary and secondary TezRs are localized extracellularly and are DNA- and RNA-based, they could potentially be overlooked within the whole set of the effects of extracellular nucleic acid destruction in microbial communities on antibiotic susceptibility. Indeed, it is a well-documented observation that extracellular nucleic acids in bacterial biofilms contribute to antibiotic resistance and their destruction by nucleases, including those produced by different bacteria at the site of infection, can increase the efficacy of antibiotics against microbial biofilms [130,131,132]. There are several explanations for the mechanisms by which nucleases enhance the effect of antibiotics based on the role of extracellular nucleic acids in bacterial adhesion and biofilm formation and their role in horizontal gene transfer [133]. Considering the nucleic acid-based structure of primary and secondary TezRs, it is worth noting that at least some of the observed effects of nucleases on microbial biofilms could be associated with the loss of TezRs.

Destruction of TezRs following treatment with nucleases triggers profound transcriptome alterations in bacteria. Since many bacteria produce extracellular nucleases during real-life infection within polymicrobial communities, the resulting destruction of TezRs might either upregulate or downregulate antibiotic resistance genes [134,135]. Moreover, the combined loss of DNA- and RNA-based TezRs with the formation of “drunk cells” has been shown to boost spontaneous mutagenesis, which is known to result in enhanced antibiotic resistance [136,137].

Therefore, the lack of consideration of polymicrobial-type bacterial growth by routine ASTs ignores the receptive and regulatory roles of TezRs in antibiotic resistance, resulting in both false-positive and false-negative antibiotic selection.

### 4.5. Currently Unknown Pathogens Are Not Analyzed by Conventional Antimicrobial Susceptibility Tests

ASTs assay antibiotic efficacy against only well-recognized lead pathogens. However, not all bacterial pathogens have yet been identified. Currently, over 90% of bacterial species are still unculturable, meaning that the current understanding of the entire list of pathogenic bacteria is incomplete [138,139]. Recent years have witnessed the discovery of previously unknown pathogens implicated in a variety of infections and cancers [140,141,142,143,144]. Finally, some bacteria that were previously considered non-harmful, such as *Aeromonas* spp., *Prevotella* spp., and *Herbaspirillum* spp., have recently been shown to be pathogenic and to contribute to the development of different human infections, including lung and urinary tract infections [145,146]. Oversight of these bacteria as pathogenic using standard ASTs would lead to therapy failure.

A summary of the reasons for the failure of ASTs to select effective antibiotics is presented in Table 1.

## 5. Emerging Methods for Antibiotic Selection

There is an unmet need for the development of novel culture-based methods for antibiotic selection that would take into consideration complicated interbacterial interactions within microbial biofilms and maximally closely recapture the real-life interactions of antibiotics with the mixed biofilm at the site of infection. The development of more effective ASTs should consider the fundamental flaws described above for conventional ASTs. Therefore, the main desirable characteristics of emerging tests for antibiotic selection are as follows: (1) culture-based; (2) rapid and provide results within 8 h from the collection of biosamples; (3) take polymicrobial biofilms into consideration; (4) study the effects of antibiotics on previously unknown pathogens; (5) analyze antibiotic efficacy based on tissue and not plasma concentrations of antibiotics; and (6) take into consideration the type of tested antibiotics—time-dependent, concentration-dependent, or dependent on total drug exposure with the AUC mechanism of antibiotic action.

A comparison between the available AST and “ideal” that would correct the fundamental flaws of conventional tests is shown in Table 2.

Recently, novel methods for antibiotic selection that consider all of these characteristics have been developed. AtbFinder is a rapid, culture-based test based on a proprietary pure-culture-free methodology [147]. This is the first diagnostic test to examine the entire microbial response to antibiotics, and not just that of the lead pathogen. The basis of AtbFinder is a novel growth “TGV agar” that enables the growth of a diverse set of bacteria from biosamples in the form of a mixed biofilm with the formation of an extracellular polymetric matrix and surface film. Therefore, polymicrobial biofilms that grow on TGV agar resemble all features of multispecies microbial communities with the modulation of QS- and TezR-modulated antibiotic resistance. Originally, this medium was developed for the isolation of previously unculturable bacteria because it supports the growth of even the most fastidious species. TGV medium outperformed other rich media such as LB (Luria-Bertani) and Columbia agar in terms of bacterial diversity and demonstrated an increased richness with higher ACE and Chao 1 indices for bacterial growth within 4 h of post-plating of biosamples, providing visible growth for 97.5% (119 of 122) monomicrobial cultures and 100% (122 of 122) within 8 h. [140,148,149].

AtbFinder is a 48-well plate filled with novel TGV agar. Unlike conventional AST methods that utilize MIC and susceptibility breakpoints to evaluate antibiotic efficacy, AtbFinder determines antibiotic efficacy based on quantifying antibiotic penetration into different tissues [42]. Moreover, the concentration of antibiotics added, depending on their class, reflects the reliance of their action on T > MIC, Cmax/MIC, or AUC/MIC. Therefore, the agar in each well was individually supplemented with antibiotics at concentrations that could be achieved at certain sites of infection. For example, since levofloxacin penetrates the lungs and skin differently, the concentration of levofloxacin added to the agar of AtbFinder for the selection of antibiotics for the treatment of lung infections would be different from the concentration added for the selection of antibiotics for the treatment of skin and soft tissue infections.

**Figure 1:**
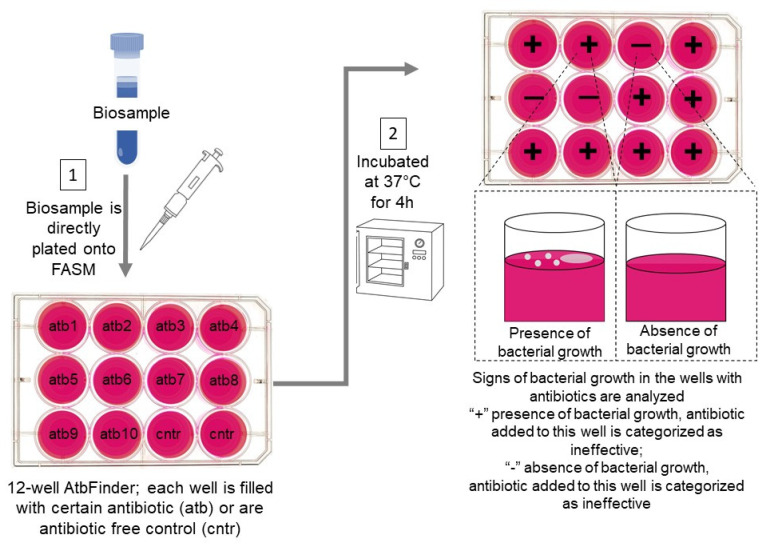
Reprinted with permission from ref. [144]. AtbFinder principle of operation and performance. An illustrated schematic of the AtbFinder performance protocol developed for different biosamples. (1) A biosample, which can be optionally diluted with sterile water, is plated onto the wells of multi-well AtbFinder plates (here, for the representation purposes we depicted a 12-well plate). The testing wells contain TGV nutrient agar supplemented with antibiotics (one or several antibiotics per well) selected as per current therapeutic guidelines and added to the medium at concentrations clinically achievable at the site of infection. Two control wells contain antibiotic-free TGV agar. (2) Plate reading is performed following sampling and incubation at 37 °C for 4 h. The presence of microbial growth is identified with the naked eye and can be confirmed with a stereoscopic microscope. Microbial growth in any testing well means that the antibiotic that has been added to the nutrient medium in this well is “ineffective”. The absence of bacterial growth in the well enables categorization of the antibiotic(s) present in the well as “effective”.

The workflow of AtbFinder is presented in Figure 1. Human biosample (sputum, wound swab or urine) is directly plated to each well of AtbFinder, thus all the organisms are grown together. The only limitation is that blood, due to its antibacterial properties needs to be additionally processed and cannot be plated directly on the TGV agar of AtbFinder. Next, AtbFinder undergoes incubation, and the presence or absence of any microbial growth is measured within 4 h.

AtbFinder has been broadly studied for antibiotic selection in both mono- and multi-species bacterial cultures. A critical parameter for any AST is accuracy with a minimal number of false-positive or false-negative results. The accuracy of AtbFinder’s performance was determined for random selection of 10 different antibiotics at 4 h and 24 h timepoints of bacterial cultivation. The correct antibiotic selection for AtbFinder was achieved after 4 h at 98.9%, and the sensitivity, specificity, positive predictive value, and negative predictive value were 99.6%, 98.1%, 98.5%, and 99.4%, respectively. Next, the same parameters were re-analyzed at 24 h to see whether a decrease in cultivation time to 4 h could contribute to error. After 24 h, the specificity, positive predictive value, and negative predictive value were 99.6%, 98.5%, 98.8%, and 99.4%, respectively. These data prove that even within 4 h of cultivation time AtbFinder was able to provide highly accurate antibiotic selection. Category agreement was also high at both 4 and 24 h. Even after 4 h, the overall category agreement for all tested antibiotics was 98.9%, the rate of very major errors was only 1.5%, and the rate of major errors was 0.6%. These values were only nominally higher if the cultivation was extended up to 24 h, with the overall category agreement for all antibiotics tested (99.1%) and the rates of very major errors (1.2%) and major errors (0.6%) [147].

In the same study, AtbFinder performance to select antibiotics against the lead bacterial pathogen directly from polymicrobial biosamples was compared to standard microbroth dilution ASTs that use pure-bacterial cultures isolated form the same polymicrobial biosamples. In 11% of cases, when antibiotics were ineffective with AtbFinder but were shown to be effective with the microbroth dilution method, the follow-up sub-cultivation of pathogens from the TGV agar showed that the lead bacterial pathogen remained and was not eliminated by the tested antibiotic. Therefore, this 11% represents an enormously high rate of false-positive results obtained by conventional ASTs, which causes the selection of ineffective antibiotics. These results are a notable demonstration of how failure to account for biofilm formation and complicated interbacterial interactions in multispecies microbial communities results in the failure of routine ASTs to select effective antibiotics.

Recently, AtbFinder was used in a pilot study to formulate an antibiotic regimen for patients with cystic fibrosis [150]. The results of AtbFinder-driven antibiotic therapy were compared to the performance of the same patients at time points when antibiotics were selected using standard AST. Antibiotics selected with AtbFinder were more effective, which resulted in a dramatic improvement in patients’ lung infections. The switch to AtbFinder-guided therapy resulted in the eradication of *P. aeruginosa* in 81% of cases. Along with *P. aeruginosa*, other respiratory pathogens, such as *S. aureus*, *Achromobacter xylosoxidans*, and *S. maltophila*, including strains exhibiting multidrug resistance, were eradicated in subjects with cystic fibrosis after antibiotic therapy was switched to drugs selected with AtbFinder.

It also completely arrested hospitalizations due to pulmonary exacerbations, even among patients who had up to three episodes of pulmonary exacerbations per year. As a result of infection reduction, the use of antibiotic selection using AtbFinder improved lung function (FEV1%) in patients with cystic fibrosis that would normally be treated with antibiotics selected with standard ASTs, is known to be constantly declining due to the progression of the infection. Importantly, following the switch of antibiotics to those selected with AtbFinder, the total number of systemic antibiotic courses was reduced by twofold with a trend of reducing the use of broad-spectrum antibiotics. This means that the improvement of subjects was due to the fact that AtbFinder selected more effective antibiotics, and not because more antibiotics or broad-spectrum antibiotic courses were used.

AtbFinder can be customized for various sites of infection, based on the antibiotics and their concentrations that are included in the panel. Recently AtbFinder with the panel of antibiotics used for the treatment of urinary tract infections (UTI) added to TGV agar at concentrations that can be achieved in urea, was successfully used to identify effective antibiotics against recurrent infection [151]. In the compassionate use study, antibiotics selected with AtbFinder eradicated recurrent UTIs that were previously unsuccessfully treated with multiple antibiotic courses.

In summary, studies using AtbFinder show how novel tests that fix different flaws of conventional ASTs can improve the efficacy of antibiotic therapy.

## 6. Conclusions

The lack of effective strategies to combat bacterial infections is alarming in both developed and developing countries. With only a few novel antibiotics being registered during the last two decades and the global spread of antibiotic resistance, the failure of conventional ASTs to select effective antibiotics is particularly concerning. Many underlying causes contribute to the lack of novel antibiotics and ARG spread, and it would take decades to solve these problems. However, finding more effective strategies for antibiotic selection can be regarded as a new holy grail for assessing antibiotic resistance that can shortly become available. This is particularly important for lung, skin, soft tissue, and urinary tract infections because conventional ASTs select antibiotics without taking into account the particularities of antibiotic penetration in non-blood tissues.

Today, standard culture-based ASTs are the gold standard for antibiotic selection, but along with a lack of accuracy to predict the in vivo success or failure of antibiotic therapy, they take too long to deliver results [152]. A number of available methods based on DNA and RNA sequences can accelerate antibiotic selection by eliminating pure-culture isolation, but they also have a number of critical shortcomings. Both culture-based and genetic-based AST have several fundamental drawbacks originating from the outdated and inaccurate belief that human infections are monobacterial. These shortcomings result in 50% of all therapies for infections starting with sub-optimal and even ineffective antibiotics [13].

Novel methods that account for the typical shortcomings of current ASTs are urgently required. These novel tests have the potential to unlock the untapped potential of already available antibiotics, bringing the treatment of deadly infections to a new level of efficacy.

## Figures and Tables

**Table 1:** Major mechanisms underlying the failure of conventional antimicrobial susceptibility tests (ASTs) to select antibiotics for non-blood-born bacterial infections.

Important Considerations Overseen by Conventional ASTs	Results of the Lack of Consideration by Conventional ASTs
Real-life antibiotic contraptions at the site of infection	Use of MIC and susceptibility breakpoints based on pharmacokinetic/pharmacodynamic (PK/PD) of antibiotics in blood by conventional AST is misleading, since concentrations of antibiotics in different tissues differ from those in blood
	Modulation of time-dependent, concentration dependent, and dependent on total drug exposure with the area under the curve (AUC) mechanism of antibiotic action
Biofilm type of growth	Misses the effects of surface membrane like layer and extracellular polymeric substances on antibiotic tolerance
	Persister tolerance
	Altered antibiotic response of cells with reduced metabolic activity
	Effects of quorum sensing on antibiotic response
	Effects of TezRs receptors on antibiotic response
Polymicrobial type of infection	Unique transcriptomic activity of antibiotic resistance genes (ARG) within polymicrobial community
	Collective response to antibiotics
	Mutual food addiction
	Role of “supporting bacteria” or “accessory pathogen” in antibiotic response of the lead pathogen
Accounting for unknown pathogens	The role of currently unknown pathogens in infection

**Table 2:** Comparison between “ideal” methods for the selection of antibiotics and those employed by existing antimicrobial susceptibility tests (ASTs).

Characteristics	Ideal AST	Culture-Based AST	Rapid AST for Pure Cultures	Nucleic Acid-Based Amplification
Recaptures environment at the site of infection	Yes	No	No	Yes
Confirmation of antibiotic resistance	Yes	No	No	No
Works without need for pure bacterial culture	Yes	No	No	Yes/No
Time from biosample to results (h)	<8	48–120	30–40	2–8
Places patient on guided antibiotic therapy at day 1	Yes	No	No	Yes
Appropriate for fastidious/unknown bacteria	Yes	No	No	Yes/No
Accounts for biofilm-type of growth	Yes	No	No	No
Accounts for modulation of antibiotic response of lead pathogen by other bacteria at the site of infection	Yes	No	No	No
Accounts for not-yet culturable bacteria	Yes	No	No	Yes/No
Considers antibiotics’ PK/PD in different organs	Yes	No	No	No
Accounts for collective response to antibiotics	Yes	No	No	No
Accounts for activity of genes of antibiotic resistance	Yes	No	No	No
Slow growth and persisters	Yes	No	No	No
Antibiotic stewardship	Should reduce the number of used antibiotics	Neutral	Neutral	Increased antibiotic use

## Data Availability

Not applicable.
